# The prevalence and profile of autism in individuals born preterm: a systematic review and meta-analysis

**DOI:** 10.1186/s11689-021-09382-1

**Published:** 2021-09-21

**Authors:** Catherine Laverty, Andrew Surtees, Rory O’Sullivan, Daniel Sutherland, Christopher Jones, Caroline Richards

**Affiliations:** 1 School of Psychology, University of Birmingham, Birmingham, B15 2TT UK; 2 Forward Thinking Birmingham, Birmingham Women’s and Children’s NHS Foundation Trust, Birmingham, UK; 3 School of Psychology, Loughborough University, Loughborough, LE11 3TU UK

**Keywords:** Autism, Prematurity, Meta-analysis, Preterm, Low birth weight

## Abstract

**Introduction:**

Preterm birth (<37 weeks) adversely affects development in behavioural, cognitive and mental health domains. Heightened rates of autism are identified in preterm populations, indicating that prematurity may confer an increased likelihood of adverse neurodevelopmental outcomes. The present meta-analysis aims to synthesise existing literature and calculate pooled prevalence estimates for rates of autism characteristics in preterm populations.

**Methods:**

Search terms were generated from inspection of relevant high-impact papers and a recent meta-analysis. Five databases were searched from database creation until December 2020 with PRISMA guidelines followed throughout.

**Results:**

10,900 papers were retrieved, with 52 papers included in the final analyses, further classified by assessment method (screening tools *N*=30, diagnostic assessment *N*=29). Pooled prevalence estimates for autism in preterm samples was 20% when using screening tools and 6% when using diagnostic assessments. The odds of an autism diagnosis were 3.3 times higher in individuals born preterm than in the general population.

**Conclusions:**

The pooled prevalence estimate of autism characteristics in individuals born preterm is considerably higher than in the general population. Findings highlight the clinical need to provide further monitoring and support for individuals born preterm.

**Supplementary Information:**

The online version contains supplementary material available at 10.1186/s11689-021-09382-1.

## Introduction

Preterm birth is defined as birth occurring at a gestational age of less than 37 weeks [1] and accounts for 15 million births worldwide each year [2]. Many infants born preterm experience immediate and significant health complications, which often lead to extended periods of hospitalisation on neonatal units [3]. Longitudinal research demonstrates that even babies born preterm who *do not* present with immediate health complications show a significant increased likelihood for later adverse neurodevelopmental outcomes such as intellectual disability [4, 5]. This increased likelihood perseveres across the lifespan, with employment rates, educational qualifications and socioeconomic status negatively impacted into adulthood [6]. As a result of medical and technological advances, survival rates for infants born preterm are rising [7]. It is therefore of growing importance to quantify the neurodevelopmental trajectory of children born preterm.

Preterm birth is associated with heightened rates of autism when compared to birth that occurs at term [8, 9]. Autism is characterised by impairments in social communication and social interaction, and restricted patterns of repetitive behaviour [2, 10, 11]. Advances in understanding of subtle and nuanced manifestations of autism now mean that individuals can be identified earlier, with diagnoses as early as 2 years old shown to be stable over time [12]. Advances in research also highlight that individuals may display difficulties in isolated areas comparable to those with autism, yet do not meet the criteria for formal diagnosis [13]. Considering the presence of these broader autism characteristics or an atypical autism phenotype is therefore imperative when aiming to meet individual needs regarding support and service use.

A notable increased prevalence of autism has been identified for those born very preterm (4–6%) [14]. Lower birth weight, earlier gestational age at birth and male gender have been associated with heightened rates of autism in preterm samples [15]. Historically, clinical resources were focussed towards those very preterm births (<32^+0/7^ weeks), who represent around 10% of all preterm births [16], with infants born closer to the term often considered to be as biologically mature as term-born infants [17]. Recent literature has documented a shift in understanding the needs of those born moderately-late preterm (32–36 weeks of gestation), who represent the majority of all preterm births [18]. Although children born moderate-late preterm often present without immediate medical complications, they are still at greater risk for adverse neurodevelopmental outcomes and even at greater risk of infantile mortality than those born at term [19]. In an attempt to recognise the difficulties which those born closer to term experience, the definition ‘near term’ was changed to ‘late preterm’ to acknowledge that infants born closer to term are still at heightened risk, with clinicians now considering gestational age and subsequent increased likelihood as a continuum [20]. It is therefore important that the synthesis of the current literature regarding neurodevelopmental outcomes reflects this and considers outcomes *across* gestational age without being limited to those born at the earliest gestations.

A previous meta-analysis examined studies identifying the prevalence of children who met clinical cut-off for autism using comprehensive diagnostic assessments [9]. A pooled estimate of 7% was returned. This strategy was important in identifying the rates of preterm children who are likely to obtain diagnoses. It does not, however, address three vital concerns: (i) studies using diagnostic assessments have tended to focus on very preterm samples at the expense of moderate-late preterm samples. Current literature shows a clear bias within assessment methods and population samples, in which diagnostic assessments are used more in very preterm populations [21, 22], whereas screening measures are utilised more in moderate-late preterm groups [23]. (ii) Studies using clinical cut-off on diagnostic assessments may miss children born preterm who show atypical presentations. Research suggests prematurity may impact development through distinct pathways, leading to distinct behavioural phenotypes and potentially alternate profiles of autism behaviour [24]. (iii) Studies using diagnostic assessments provide no quantification of those children who show sub-threshold difficulties with social communication, interaction and restricted repetitive behaviours. Screening tools are used more commonly in preterm populations as a method of risk stratification that is both cost and time effective [25]. Addressing these concerns is vital to ensure that prevalence rates are better understood in (i) *all* children born preterm, (ii) children with *atypical presentations* and (iii) children whose traits may cause significant difficulties, but may fall short of full diagnostic criteria identified via screening tools.

In summary, heightened rates of autism have been identified in individuals born preterm. While methods of early identification in high-risk samples have become more reliable, the precise profile of autism characteristics in individuals born preterm is not well documented. Most recent prevalence rates confirm that preterm populations are high-risk groups, yet meta-analytic approaches have excluded screening measures, meaning they have focussed mainly on very preterm groups, and those with the most typical and severe presentations. Therefore, the present systematic review and meta-analysis aim the following:

i. Synthesise existing literature and calculate pooled prevalence estimates for autism based on diagnostic and screening measures of autism characteristics in preterm samples.
ii. Compare pooled prevalence estimates in the preterm population with estimates of autism in the general population
iii. Identify participant characteristics that may be associated with autism characteristics in preterm samples

## Method

### Search strategy

Before a search was undertaken, the study was preregistered on PROSPERO (Available at: https://www.crd.york.ac.uk/prospero/display_record.php?ID=CRD42019125412). Search terms were generated from inspection of relevant high-impact papers and recent meta-analyses (See Table 1). Additionally, we hand-searched a recent meta-analysis to identify further publications [9].

**Table 1:** Search terms used to search Ovid MEDLINE, Ovid PsychINFO, Ovid Embase, Ovid Embase Classic and PubMed from the beginning of creation to late January 2020

Search terms	
#1 Autism	“autis*”, “autism*”, “autistic*”, “ASD”, “autism spectrum disorder*”, “PDD-NOS”, “PDDNOS”, “ unspecified PDD”, “pervasive developmental disorder*”, “pervasive developmental disorder not otherwise specified”, “Asperger*”, and “Asperger* syndrome”. The premature search terms included premature*”, “preterm”, “prematur*”, “low birth weight”.
#2 Preterm	“premature*”, “preterm”, “prematur*”, “low birth weight”.

### Study selection

**Fig. 1:**
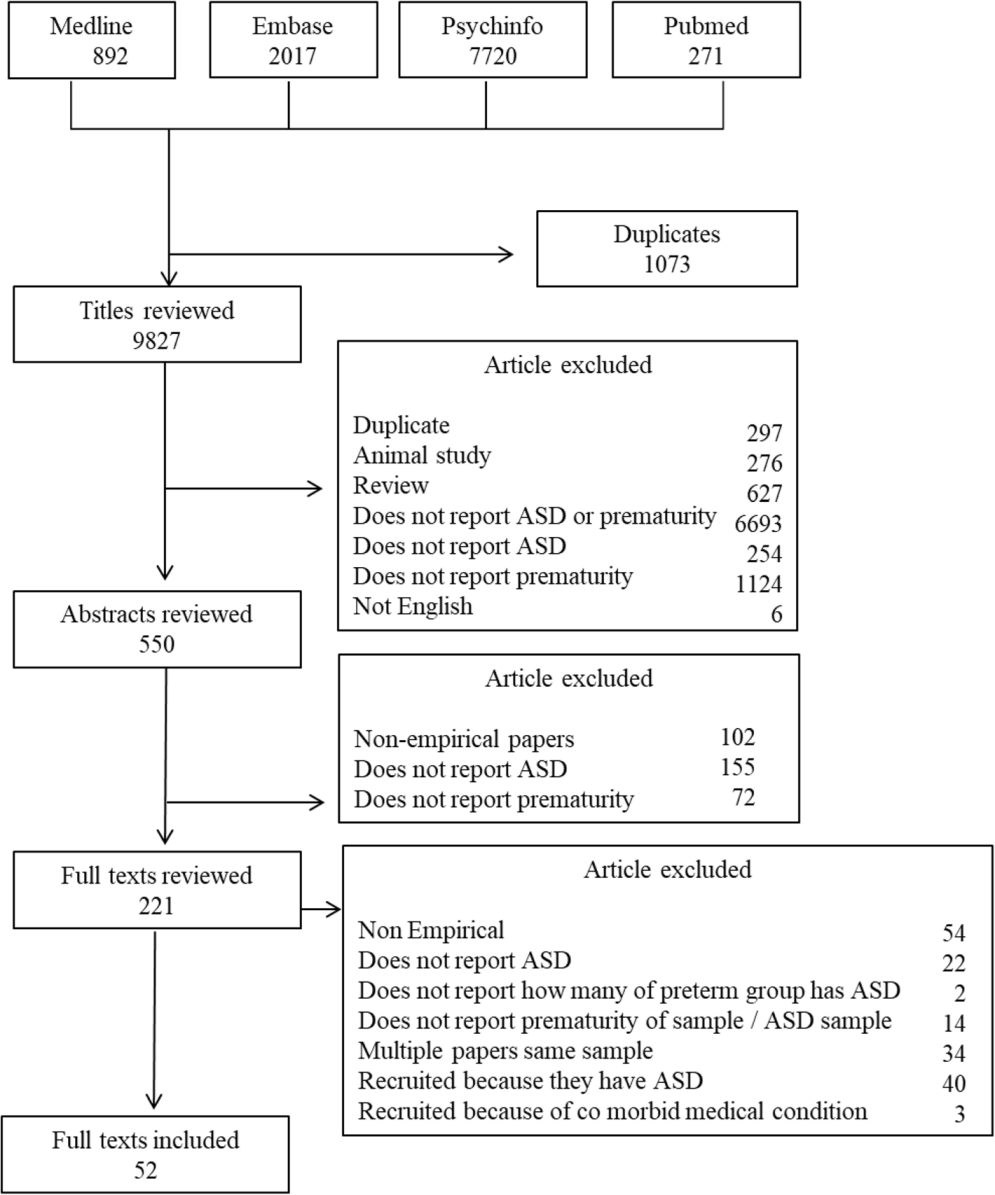
A flow chart detailing papers included and excluded at each stage of screening and review

Initial searches returned 10,900 responses that were systematically assessed for suitability and inclusion (Fig. 1). An initial automatic ‘de-duplication’ process was run using EndNote software, with the corresponding researcher then manually inspecting for any that were missed. Papers were then assessed in three stages. For the first stage of selection, we used predefined inclusion and exclusion criteria to assess the titles/abstracts for inclusion. In the second stage, we reviewed papers against stage two criteria in full text (see Supplementary Table 1 & Supplementary Table 2 in supplementary materials for additional information). Finally, for the third stage, we rated all included papers against a multi-level quality matrix described below.

To eliminate the risk of researcher bias, two additional researchers screened a subsample of papers at both stages 1 and 2 outlined above (19%). They then completed quality ratings against the quality weighting framework for *all* included papers. A good level of reliability between the two independent researchers was obtained (weighted Cohen’s Kappa 0.7). Where discrepancies were identified, an agreement was made between the two raters.

### Quality criteria

The quality of all studies that progressed to stage three of the review process was assessed using standardised quality weighting criteria to control for threats to validity. The quality effects model extends the random-effects model, allowing for papers rated as higher quality to be given more weight in estimates of prevalence three key areas were assessed; autism assessment, sample identification and study design (Table 2).

**Table 2:** Quality Criteria for autism Assessment, Sample Identification and Study Design

	0 – Poor	1 – Adequate	2 – Good	3 - Excellent
**Autism Assessment**	Not specified / reported Clinical judgement only	Informant report / self-report instrument Screening instrument Clinical judgement against specified diagnostic criteria (DSM-5 or ICD 10)	Diagnostic instrument / interviews -	Consensus from multiple assessments, including at least one diagnostic instrument
**Sample Identification**	Not specified / reported	Single restricted or non-random sample (specialist clinic or previous research study)	Multiple restricted or non-random samples (multi-region specialist clinics)	Random or total population sample
**Study Design**	Not specified / reported	Case series	Historically identified cohort (e.g., via patient records)	Prospective cohort

A visual matrix of quality weighting is presented in Table 3, alongside study characteristics and outcome data. From each paper, data were then extracted and the number of participants meeting cutoff for an autism diagnosis was taken.

**Table 3:** Sample characteristics and quality criteria for included papers

Author	Location	Gestational age of sample	Diagnostic tools used	Sample Size	No. preterm individuals scored over the autism threshold	Quality Criteria		
						Autism	Sample Identification	Design
Abolfotouh et al, 2018 [26]	Saudi Arabia	22-23wks (10)	Denver developmental screening test	63	4 (Hyperactive autistic)	1	1	3
		24-25wks (43)						
		26-28wks (52)						
		29-30wks (12)						
Al-Hathlol et al.*, 2020* [27]	Saudi Arabia	< 32 weeks or < 1500 g	Unspecified questionnaire	158	3	1	1	2
Atladottir et al, 2016 [28]	Denmark	24–43 weeks	Diagnosis was retrieved from the Danish Psychiatric Central Register	82,911	1203	1	3	2
Bakian et al, 2018 [29]	Utah	< 37 weeks	Registry of Autism and Developmental Disabilities	4855	112 (ASD)	1	2	2
Boone et al, 2018 [30]	Columbus	< 30 weeks	PDDST-II-DCS	528	190	3	2	3
			ADOS-2	555	24 (ASD)			
Bröring et al, 2018 [31]	Amsterdam	Very preterm (30.2 mean)	SRS, Children’s Communication Checklist, SCQ	57	0	1	1	3
Brumbaugh et al.*, 2020* [32]	Minnesota	< 37 weeks	Medical and educational records	7876	266	1	3	3
Chen et al.*, 2020* [33]	Taiwan	< 32 weeks or < 1500 g	ADOS and ADI-R	324	30	3	2	3
De Groote et al, 2006 [34]	Belgium	< 37 weeks	Autism Diagnostic Observation Schedule-Generic	25	2 (Autism)	2	1	3
De Oliveira Holanda et al.*, 2020* [35]	Brazil	< 37 weeks	MCHAT	40	20	1	1	3
Dudova et al*,* 2014 [36]	Prague	NR	M-CHAT	157	28	3	2	3
			ADOS	33	15			
Gray et al*,* 2015 [37]	Brisbane	very preterm	MCHAT, The CBCL & DASS	97	13 (Autism)	1	1	3
Guy et al, 2015 [23]	East Midlands	32–36 weeks	MCHAT & follow up phone interview	634	92	1	2	3
Hack et al*,* 2009 [38]	America	26 weeks	Parent Child Symptom Inventory	219	8	1	1	2
Harel-Gadassi et al., 2018 [39]	Jerusalem, Israel	31.16 (mean)	M-CHAT	93	25	3	1	3
			ADOS-T	101	8			
Hubert et al.*, 2020* [40]	Poland	GA Mean 27.8	Childhood autism spectrum test	89	5	1	1	3
Hvidtjorn et al.*, 2011* [41]	Denmark	< 37	Public child mental health service	37,283	277	1	2	3
Hwang et al, 2013 [42]	Taiwan	Late preterm = 1078, Later preterm = 28,947, Full-term = 1,104,071	Coded by doctors based on ICD-9-CM	Early preterm = 1078, Later preterm = 28,947	Early preterm = 24, Later preterm = 387	1	2	3
Ikejiri et al*,* 2016 [43]	Juntendo	> 33 Weeks	DSM-4-TR	59	9 (ASD)	1	1	3
Indredavik et al.*, 2004* [44]	Norway	GA mean: 28.8	Interviewed and conclusions drawn according to DSM	56	1	1	2	3
Johnson et al*,* 2010 [45]	UK & Ireland	< 26	SCQ	189	29 (ASD)	1	3	3
Johnson et al*,* 2018 [24]	England	32 wks = 38	MCHAT	638	92	1	2	3
Joo et al.*, 2015* [46]	Korea	24–36	CARS	58	1	2	2	3
Kihara et al.*, 2015* [47]	Japan	GA mean: 27.4	Clinical assessments & DSM criteria	321	35	1	2	3
Klimek et al., 2018 [48]	Poland	28 weeks (Mean)	The Childhood Autism Spectrum Test	86	5	1	1	3
Kuban et al 2009 [49]	US	before 28 weeks gestation	MCHAT	988	212	1	2	3
Kuzniewicz et al*,* 2014 [50]	California	< 24 weeks	ASD evaluation centre	15,696	280	3	2	3
Laerum et al., 2019 [51]	Norway	28.9 weeks (mean)	Autism Spectrum Quotient	59	21	1	1	3
Lean et al.*, 2020* [52]	USA	< 30 weeks	ADOS & Parent report	85	11	3	1	3
Leavey et al, 2013 [53]	California		Diagnostic codes	33,121	213	1	2	2
Lederman et al, 2018 [54]	São Paulo, Brazil	29.5 (mean)	M-CHAT & Autism Behaviour Checklist	60	4	3	1	3
Limperopoulos, et al, 2008 [15]	Boston	NR	MCHAT	91	23	1	1	3
Matheis et al, 2018 [55]	Louisiana	< 37 Weeks	BISCUIT Part 1	687	213	1	2	2
Mir et al.*, 2020* [56]	Texas	< 28 weeks	MCHAT	218	31			
			ADOS & CARS	218	16			
Mohammed et al.*, 2016* [57]	Saudi Arabia	GA 27–33	Clinical assessments & DSM	107	5	1	1	3
Moore et al, 2012 [25]	England	NR	MCHAT	523	216 (Positive result on the MCHAT Autism)	1	3	3
Nagai et al.*, 2020* [58]	Japan	VLBW < 1500	DSM-5 and ADOS	38	10	3	1	3
Persson et al.*, 2020* [59]	Norway	< 37 weeks	Medical records	165,845	3544	1	3	2
Pineda et al.*, 2014* [60]	USA	< 30 weeks	MCHAT	77	19	1	2	2
Pinto-Martin et al.*, 2011* [21]	New Jersey	GA mean:31.2	ADI-R/ADOS	623	14	2	2	3
			SCQ	623	117			
Pritchard et al*,* 2016 [61]	Australia	< 29 Weeks	ADOS-G	15	3	2	1	3
			M-CHAT	169	22			
Rand., et al. 2016 [62]	New Zealand	< 32	DAWBA	102	3	1	2	3
Rutkowska*,* et al, 2018 [63]	Poland	< 28 Weeks	Screening Tool for Autism in Toddlers & Young Children	10	4	1	1	1
Sharp et al.*, 2018* [64]	Australia	22–24 wks	Multidisciplinary team assessment	159	9	1	1	2
Stephens et al, 2012 [65]	NICHD Neonatal Research Network	< 27 weeks	PDDST-II & adapted items from the ADOS	554	Positive screen - 113	2	2	3
Sumanasena et al.*, 2018* [66]	Sri Lanka	< 34	DSM Criteria	39	3	1	2	3
Treyvaud et al, 2013 [14]	Melbourne Australia,	< 30 weeks	DAWBA	177`	8 (ASD)	1	1	3
Twilhaar et al., 2019 [67]	Amsterdam, Netherlands	29.2 weeks (mean)	Social Responsiveness Scale	60	18	1	1	3
Verhaeghe et al*,* 2016 [68]	Belgium	Before 27 weeks	SRS	47	21	3	2	3
			ADOS, and The ADI-R	43	14			
Vermeirsch et al.*, 2020* [69]	Belgium	< 30 weeks	SRS	55	22			
			ADOS, ADI-R & clinical information	55	7			
Yaari, et al, 2016 [70]	Israel	24–34 weeks	The AOSI and ADOS-T	99	High ASD risk – 8, Low ASD risk - 91	–	–	–
Yang et al.*, 2015* [71]	Taiwan	Mean BW 1200 g	Diagnostic tools, observations and parental reports	61	2	1	2	3
You et al., 2019 [72]	China	35.5 weeks (mean)	M-CHAT	102	9	1	2	3

### Statistical analysis

All estimated prevalence rates for autism in preterm samples were extracted from papers remaining in the final stage of review. These estimates were collated based upon the type of assessment tool used; screening tools or diagnostic assessments. These data were analysed to generate two pooled prevalence estimates, with random- and fixed-effects models created for both. Fixed-effects models assume equal weighting of studies, with any error attributed to sampling error, whereas random-effects models allow the true effect to vary between studies, with weighting fluctuating between studies [73]. To calculate the random-effects model within the current study, the restricted maximum-likelihood estimator was used. This estimator is more robust than traditional DerSimonian-Laird estimates in non-normal distributions of effect, as the method restricts the likelihood estimates to control for underestimation and minimise bias [74]. This decision was supported from analysing the Quantile-Quantile plots (see Supplementary Figure 1 in supplementary materials), which suggested the fixed-effects model did not conform to normal distribution. A quality effects model (QEM) for each assessment method was also produced to assess the impact of methodological variation as defined and weighted by the quality framework outlined above, and this quality effects estimate was compared to the random-effects estimate (see Supplementary Figure 2 in supplementary materials). In order to explore the prevalence of autism amongst individuals born preterm in comparison to rates amongst the general population, odds ratios (OR) with 95% CI were generated. This analysis compared the random-effects pooled prevalence estimates from diagnostic assessment methods with the most recent total population surveillance prevalence estimate for autism diagnosis (one in 54) [75]. This particular population surveillance was chosen given its use of gold standard assessments and diagnostic and statistical manual definitions to confirm the diagnosis. Data from this paper were also referenced by the CDC and others widely support the conservative estimate of 1 in 54 as a representative and inclusive population estimate.

## Results

### Prevalence of autism characteristics in individuals born preterm

**Fig. 2:**
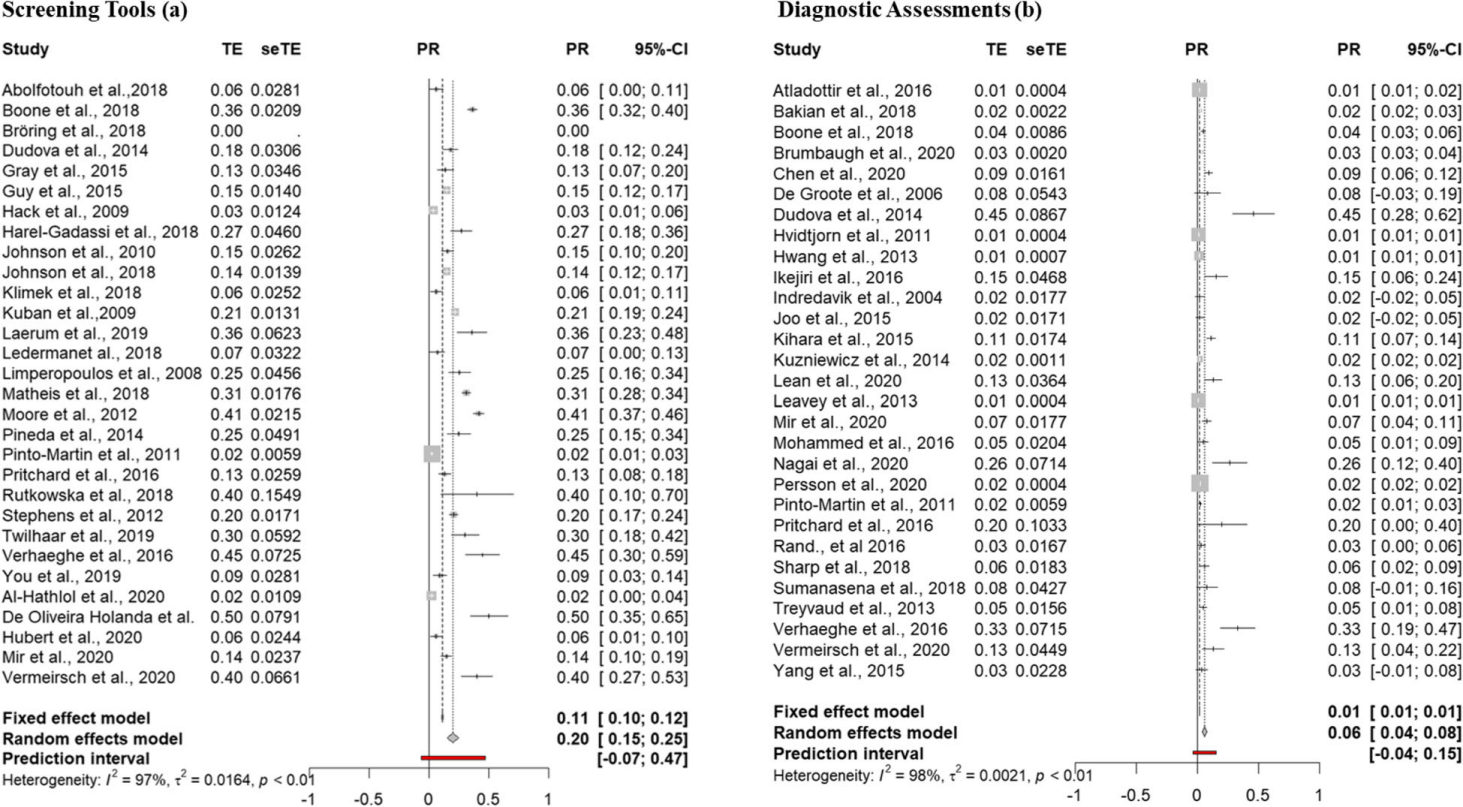
Fixed- and random-effects pooled prevalence estimates for autism characteristics in individuals born preterm using screening and diagnostic tools

52 studies were included in the final meta-analysis; 23 screening tools only, 22 direct assessment only and 7 both. Pooled prevalence estimates of autism using random- and fixed-effects models were generated for both screening tools and diagnostic assessment (See Figure 2).

#### Screening tools

The fixed-effects model generated a weighted prevalence estimate of 11% (*z* = 31.89, *p* = <.001; 95% CI 0.1018; 0.1151%) for autism characteristics in individuals born preterm. The random-effects model generated a prevalence estimate of 20% (*z* = 7.97, *p* = <.001; 95% CI 14.98; 24.75%). The random-effects model extended with a quality weighting estimated a prevalence of 21% (*z* = 8.39, *p* = <.001; 95% CI 16.38; 26.37%).

#### Diagnostic assessments

The fixed-effects model generated a weighted prevalence estimate of 1% (*z* = 73.54, *p* = 0; 95%, CI 1.36; 1.43%) for autism characteristics in individuals born preterm. The random-effects model generated a prevalence estimate of 6% (*z* = 5.8, *p* = <.001; 95% CI 3.74; 7.57%). The random-effects model extended with a quality weighting estimated a prevalence of 9% (z = 8.17, p = <.001; 95% CI 7.19; 11.74%).

In summary, the prevalence of autism characteristics was explored in individuals born preterm for both screening tools and diagnostic assessments in turn, with estimates ranging across the models created. Both fixed-effects models revealed high levels of heterogeneity (*I*^2^ = 97.3–97.8%) indicating that the fixed-effects model is not appropriate given it could not be concluded that studies were conducted under similar conditions. The random- and quality-effects models that account for variability between studies and quality respectively produced estimates between 6 and 21% (screening tools; random-effects model - 20%; quality effects model - 21%; diagnostic assessments; random-effects model - 6%; quality effects model - 9%).

##### Sources of heterogeneity

Analyses investigating influential studies did not identify any differences associated with increased prevalence rates (see Supplementary Figure 3 in supplementary materials).

##### Publication bias

Visual inspection of funnel plots created to detect publication bias showed a broad symmetrical distribution. Given the subjective nature of the visual inspection, linear regression analysis was conducted to statistically test for asymmetry [76]. A non-significant result (*p* = 1.494) suggested no evidence of publication bias, exaggerated estimates of smaller studies or the inclusion of poor quality studies.

### Comparing pooled prevalence estimates in the preterm population with estimates of autism in the general population

To explore the prevalence of autism amongst individuals born preterm in comparison to rates amongst the general population, odds ratios (OR) with 95% CI were generated. In the case of diagnostic assessments, OR analysis suggested the odds of an autism diagnosis were 3.3 times higher in individuals born preterm than in the general population (95% CI 0.24–47.60).

### To identify participant characteristics that may be associated with autism characteristics in preterm samples

To explore participant characteristics associated with autism characteristics, the influence of participants’ age at assessment and gestational age on prevalence rates were assessed using a meta-regression (Table 4). Both meta-regressions revealed no significant associations between age at assessment (screening tools, *p* =.118; diagnostic assessment, *p* =.579) or mean gestational age (screening tools, *p* =.192; diagnostic assessment, *p* =.579) and prevalence of autism characteristics. Due to insufficient data, it was not possible to assess the profile of autism characteristics in individuals born preterm and consider how this differed from individuals born at term.

**Table 4:** Participant characteristics influencing the prevalence of autism diagnosis in individuals born preterm

Covariate	Estimate	S.E.	***Z***	***p***	Lower 95%CI	Upper 95%CI
Screening tools						
Age at assessment	−0.00010	0.0005	−1.9172	.0552	−0.0021	0.0000
Gestational age	−0.0080	0.0144	−0.5546	.5791	−0.0362	0.0202
Diagnostic tools						
Age at assessment	−0.00005	0.0004	−1.3022	.1928	−0.0012	0.0002
Gestational age	−0.0175	0.0112	−1.5602	.1187	−0.0394	0.0045

## Discussion

The prevalence of autism characteristics in individuals born preterm was systematically reviewed and meta-analysed. This was the first study to meta-analyse data across both diagnostic and screening tools. Due to the inclusion bias present in studies using diagnostic assessments, this meta-analysis therefore provides novel estimates of prevalence across gestational age. The use of a robust and standardised search strategy improves the accuracy of estimates, with stringent inclusion and exclusion criteria considerably enhancing the internal validity of findings. Overall, autism characteristics were estimated to have a prevalence rate of between 6 and 20% for individuals born preterm, dependent upon assessment method used. The odds of an autism diagnosis were 3.3 times higher in individuals born preterm than in the general population. Both meta-regressions revealed no association between age at assessment or gestational age and autism characteristics in preterm samples. Results have significant clinical importance for both individuals born preterm and the provision of services.

Autism characteristics were present in 6–20% of preterm individuals in the current study. This is considerably higher than general population estimates (1.5%; [75]) and consistent with a recent meta-analysis of autism in preterm individuals that included only diagnostic tools (7%; [9]). The current study confirms and extends the findings that preterm individuals are more likely to have difficulties consistent with an autism diagnosis and also show heightened rates of autism characteristics compared to the general population.

The inclusion of data from papers that used screening tools is a key strength of the current study, as these prevalence estimates capture a broader range of autism characteristics. One consideration when comparing screening measures to diagnostic assessments is that the differing tools are perhaps asking different questions; one surrounding the presence and levels of autism characteristics while the other only identifies those that reach diagnostic cutoff. It has previously been suggested that screening measures are not as valid as diagnostic assessments [77], over-identifying the individual likelihood of autism [78]. Nevertheless, screening measures are increasingly used to help reduce clinical waiting lists and enable earlier access to support, which has been shown to improve outcomes [79]. The results from this meta-analysis suggest that 1 in 5 children born preterm warrants further assessment for autism, and as such, future research should quantify the extent to which screening tools within preterm samples generate prevalence estimates similar to diagnostic tools through the consideration of their psychometric properties.

High levels of heterogeneity were identified during analysis (*I*^2^ = 97.3–97.8%). A potential reason for the high heterogeneity could be the variability in both sample identification and populations studied. Studies with poorly identified samples (inclusive of all gestational ages) provided lower prevalence estimates of autism characteristics, while studies with well-defined samples consistently comprised individuals born very preterm, where rates of autism characteristics identified are notably higher than those born closer to term [80]. This has wide implications for future research; studies of neurodevelopmental outcomes should adhere to classifying groups using standard definitions of prematurity and consider these outcomes across all gestational ages. While samples continuously use differing definitions of prematurity, the ability to assess these groups robustly decreases.

To explore the influence of gestational age on prevalence estimates of autism characteristics, both meta-regressions were conducted on available data. No significant associations were found between the gestational age of participants and autism characteristics. Previous research has highlighted an increase in autism prevalence amongst those born very preterm when compared to those born closer to term [45, 61]. Although the results of the current analysis suggest no significant difference, caution must be taken with interpreting this result. A noteworthy limitation of this analysis was the lack of power and clarity in sample descriptions reporting effects across included studies. As mentioned previously, the most well-defined populations are often those born very preterm, with most of the current studies simply providing a mean gestational age. It is vital future research clearly defines participant groups so that outcomes can be rigorously stratified.

Further analysis was conducted to explore any mediating effect age of assessment had upon autism prevalence estimates, with no significant associations found. Research highlights the reliability of diagnosis as young as 24 months [81], and the beneficial impact this can have upon service use and support [82], the current study did not identify a significant difference in prevalence based upon the age of assessment, although more research would be needed to confidently rule out any effect of age. While this could suggest the stability of autism across the lifespan, there was again a large amount of missing data surrounding the age of participants at assessments. Research has highlighted the importance of understanding the early behavioural phenotype of preterm infants, as early behaviours and increased likelihood could all result in a later diagnosis of autism [70]. In line with World Health Organization (WHO) guidance on routine early medical and developmental follow-up after preterm birth [83], and with evidence that poorer outcomes can be identified throughout the lifespan, it is important future research focuses on providing assessments and intervening at a younger age.

Due to insufficient data, it was not possible to assess the profile of autism characteristics in individuals born preterm and consider how this differed from autism characteristics in individuals born at term. This highlights a substantial gap in the current literature, as subscale scores for social communication and restricted repetitive domains are consistently not reported, despite diagnostic and screening tools providing these outputs. While many studies utilised standardised assessment methods that are well validated, the omission of reported subscale scores significantly impacts any conclusions that can be drawn from them. Recent literature highlights that individual characteristics and traits (often measured separately through subscales of measures) are often deemed more important than an overall ‘autism severity’ score [84]. Similarly, current interventions target specific social cognitive differences and or divergent social behaviours evidenced in individuals with autism characteristics instead of a global level [85–88]. Without precise documentation of the profile of autism evidenced by individuals born preterm, it is not yet clear if interventions created for use in term populations with autism can be successfully used for individuals born preterm.

## Conclusion

The results of this meta-analysis are of significant clinical importance. Following the publication of the WHO recommendations for improving outcomes of those born preterm, neurodevelopmental outcomes of preterm infants are considered to be of particular importance [69]. Services offered to preterm individuals are lacking, with further support deemed necessary in areas such as infant neurodevelopment as well as more specific domains such as feeding and sleeping. Current data show considerably elevated prevalence of autism characteristics in individuals born preterm; it is therefore vital that services providers reflect this increased likelihood in the support and professional follow-up they offer.

## Supplementary Information

**Additional file 1.** Supplementary analysis and figures provided for additional clarity.

## Data Availability

All data generated or analysed during this study are included in this published article.

## References

[1] Howson CP, Kinney MV, McDougall L, Lawn JE (2013). Born toon soon: preterm birth matters. Reprod Health.

[2] Eisfeld J (2014). International statistical classification of diseases and related health problems. Transgender Stud Q.

[3] Escobar GJ, McCormick MC, Zupancic JAF, Coleman-Phox K, Armstrong MA, Greene JD (2006). Unstudied infants: outcomes of moderately premature infants in the neonatal intensive care unit. Arch Dis Child Fetal Neonatal Ed.

[4] Johnson S (2007). Cognitive and behavioural outcomes following very preterm birth. Semin Fetal Neonatal Med.

[5] Soleimani F, Zaheri F, Abdi F. Long-term neurodevelopmental outcomes after preterm birth. Iran Red Crescent Med J. 2014;16. 10.5812/ircmj.17965 PMC4102985 25068052

[6] Bilgin A, Mendonca M, Wolke D (2018). Preterm birth/low birth eight and markers reflective of wealth in adulthood: a meta-analysis. Pediatrics..

[7] Saigal S, Doyle LW (2008). An overview of mortality and sequelae of preterm birth from infancy to adulthood. Lancet.

[8] Singh GK, Kenney MK, Ghandour RM, Kogan MD, Lu MC (2013). Mental health outcomes in US children and adolescents born prematurely or with low birthweight. Depress Res Treat.

[9] Agrawal S, Rao SC, Bulsara MK, Patole SK (2018). Prevalence of autism spectrum disorder in preterm infants: a meta-analysis. Pediatrics..

[10] American Psychiatric Association (2014). Cautionary statement for forensic use of DSM-5.

[11] Wong HS, Huertas-Ceballos A, Cowan FM, Modi N (2014). Evaluation of early childhood social-communication difficulties in children born preterm using the quantitative checklist for autism in toddlers. J Pediatr.

[12] Moore V, Goodson S (2003). How well does early diagnosis of autism stand the test of time? Follow-up study of children assessed for autism at age 2 and development of an early diagnostic service. Autism..

[13] Happé F, Ronald A, Plomin R (2006). Time to give up on a single explanation for autism. Nat Neurosci.

[14] Treyvaud K, Ure A, Doyle LW, Lee KJ, Rogers CE, Kidokoro H (2013). Psychiatric outcomes at age seven for very preterm children: rates and predictors. J Child Psychol Psychiatry Allied Discip.

[15] Limperopoulos C, Bassan H, Sullivan NR, Soul JS, Robertson RL, Moore M (2008). Positive screening for autism in ex-preterm infants: prevalence and risk factors. Pediatrics..

[16] Blencowe H, Cousens S, Chou D, Oestergaard M, Say L, Moller AB (2013). Born too soon: the global epidemiology of 15 million preterm births. Reprod Health.

[17] Lantos JD, Lauderdale DS (2018). Late preterm birth. Preterm Babies Fetal Patients Childbear Choices.

[18] Office for National Statistics (2015). Statistical bulletin birth characteristics in England and.

[19] Frey HA, Klebanoff MA (2016). The epidemiology, etiology, and costs of preterm birth. Semin Fetal Neonatal Med.

[20] Morgan JC, Boyle EM (2018). The late preterm infant. Paediatr Child Health.

[21] Pinto-Martin JA, Levy SE, Feldman JF, Lorenz JM, Paneth N, Whitaker AH (2011). Prevalence of autism spectrum disorder in adolescents born weighing <2000 grams. Pediatrics..

[22] Joseph RM, O’Shea TM, Allred EN, Heeren T, Hirtz D, Paneth N (2017). Prevalence and associated features of autism spectrum disorder in extremely low gestational age newborns at age 10 years. Autism Res.

[23] Guy A, Seaton SE, Boyle EM, Draper ES, Field DJ, Manktelow BN (2015). Infants born late/moderately preterm are at increased risk for a positive autism screen at 2 years of age. J Pediatr.

[24] Johnson S, Waheed G, Manktelow BN, Field DJ, Marlow N, Draper ES (2018). Differentiating the preterm phenotype: distinct profiles of cognitive and behavioral development following late and moderately preterm birth. J Pediatr.

[25] Moore T, Johnson S, Hennessy E, Marlow N (2012). Screening for autism in extremely preterm infants: problems in interpretation. Dev Med Child Neurol.

[26] Abolfotouh MA, Al Saif S, Altwaijri WA, Al Rowaily MA. Prospective study of early and late outcomes of extremely low birthweight in Central Saudi Arabia. BMC Pediatr. 2018;18. 10.1186/s12887-018-1248-y PMC6106812 30134865

[27] Al-Hathlol K, Al-Obaid OM, Al-Gholaiqa TS, Al-Hathlol B, Abdulaal AE, Al-Hajress RI (2020). School performance and long-term outcomes of very preterm children conceived via in vitro fertilization. J Bras Reprod Assist.

[28] Atladóttir HO, Schendel DE, Henriksen TB, Hjort L, Parner ET (2016). Gestational age and autism spectrum disorder: trends in risk over time. Autism Res.

[29] Bakian AV, Bilder DA, Korgenski EK, Bonkowsky JL (2018). Autism spectrum disorder and neonatal serum magnesium levels in preterm infants. Child Neurol Open.

[30] Boone KM, Brown AK, Keim SA (2018). Screening accuracy of the brief infant toddler social-emotional assessment to identify autism spectrum disorder in toddlers born at less than 30 weeks’ gestation. Child Psychiatry Hum Dev.

[31] Bröring T, Oostrom KJ, van Dijk-Lokkart EM, Lafeber HN, Brugman A, Oosterlaan J (2018). Attention deficit hyperactivity disorder and autism spectrum disorder symptoms in school-age children born very preterm. Res Dev Disabil.

[32] Brumbaugh JE, Weaver AL, Myers SM, Voigt RG, Katusic SK (2020). Gestational age, perinatal characteristics, and autism spectrum disorder: a birth cohort study. J Pediatr.

[33] Chen LW, Wang ST, Wang LW, Kao YC, Chu CL, Wu CC, et al. Early neurodevelopmental trajectories for autism spectrum disorder in children born very preterm. Pediatrics. 2020;146. 10.1542/peds.2020-0297 32900877

[34] De Groote I, Roeyers H, Warreyn P (2006). Social-communicative abilities in young high-risk preterm children. J Dev Phys Disabil.

[35] De Oliveira Holanda NS, Da Costa LDO, Santos Sampaio SS, Da Fonseca Filho GG, Bezerra RB, Azevedo IG (2020). Screening for autism spectrum disorder in premature subjects hospitalized in a neonatal intensive care unit. Int J Environ Res Public Health.

[36] Dudova I, Markova D, Kasparova M, Zemankova J, Beranova S, Urbanek T (2014). Comparison of three screening tests for autism in preterm children with birth weights less than 1,500 grams. Neuropsychiatr Dis Treat.

[37] Gray PH, Edwards DM, O’Callaghan MJ, Gibbons K (2015). Screening for autism spectrum disorder in very preterm infants during early childhood. Early Hum Dev.

[38] Hack M, Taylor HG, Schluchter M, Andreias L, Drotar D, Klein N (2009). Behavioral outcomes of extremely low birth weight children at age 8 years. J Dev Behav Pediatr.

[39] Harel-Gadassi A, Friedlander E, Yaari M, Bar-Oz B, Eventov-Friedman S, Mankuta D (2018). Risk for ASD in preterm infants: a three-year follow-up study. Autism Res Treat.

[40] Hubert J, Gilarska M, Klimek M, Nitecka M, Dutkowska G, Kwinta P (2020). Small for gestational age is an independent risk factor for neurodevelopmental impairment. Iran J Pediatr.

[41] Hvidtjørn D, Grove J, Schendel D, Schieve LA, Sværke C, Ernst E (2011). Risk of autism spectrum disorders in children born after assisted conception: a population-based follow-up study. J Epidemiol Community Health.

[42] Hwang YS, Weng SF, Cho CY, Tsai WH (2013). Higher prevalence of autism in Taiwanese children born prematurely: a nationwide population-based study. Res Dev Disabil.

[43] Ikejiri K, Hosozawa M, Mitomo S, Tanaka K, Shimizu T (2016). Reduced growth during early infancy in very low birth weight children with autism spectrum disorder. Early Hum Dev.

[44] Indredavik MS, Vik T, Heyerdahl S, Kulseng S, Fayers P, Brubakk AM. Psychiatric symptoms and disorders in adolescents with low birth weight. Arch Dis Child Fetal Neonatal Ed. 2004;89. 10.1136/adc.2003.038943 PMC1721748 15321968

[45] Johnson S, Hollis C, Kochhar P, Hennessy E, Wolke D, Marlow N. Autism spectrum disorders in extremely preterm children. J Pediatr. 2010;156. 10.1016/j.jpeds.2009.10.041 20056232

[46] Joo JW, Choi JY, Rha DW, Kwak EH, Park ES (2015). Neuropsychological outcomes of preterm birth in children with no major neurodevelopmental impairments in early life. Ann Rehabil Med.

[47] Kihara H, Nakamura T (2015). Early standard development assessment characteristics in very low birth weight infants later classified with autism spectrum disorder. Early Hum Dev.

[48] Klimek M, Nitecka M, Dutkowska G, Gilarska M, Kwinta P (2018). Temperament traits in 4-year-old children born prematurely – may they suggest a threat for mental functioning?. Psychiatr Pol.

[49] Kuban KCK, O’Shea TM, Allred EN, Tager-Flusberg H, Goldstein DJ, Leviton A. Positive screening on the modified checklist for autism in toddlers (M-CHAT) in extremely low gestational age newborns. J Pediatr. 2009;154. 10.1016/j.jpeds.2008.10.011 PMC2693887 19185317

[50] Kuzniewicz MW, Wi S, Qian Y, Walsh EM, Armstrong MA, Croen LA (2014). Prevalence and neonatal factors associated with autism spectrum disorders in preterm infants. J Pediatr.

[51] Lærum AMW, Reitan SK, Evensen KAI, Lydersen S, Brubakk AM, Skranes J, et al. Psychiatric symptoms and risk factors in adults born preterm with very low birthweight or born small for gestational age at term. BMC Psychiatry. 2019;19. 10.1186/s12888-019-2202-8 PMC6636134 31315591

[52] Lean RE, Lessov-Shlaggar CN, Gerstein ED, Smyser TA, Paul RA, Smyser CD (2020). Maternal and family factors differentiate profiles of psychiatric impairments in very preterm children at age 5-years. J Child Psychol Psychiatry Allied Discip.

[53] Leavey A, Zwaigenbaum L, Heavner K, Burstyn I (2013). Gestational age at birth and risk of autism spectrum disorders in Alberta, Canada. J Pediatr.

[54] Lederman VRG, Goulart AL, dos Santos AMN, Schwartzman JS. Rastreamento de sinais sugestivos de TEA em prematuros com muito baixo peso ao nascer. Psicol - Teor e Prática. 2018;20.

[55] Matheis M, Matson JL, Burns CO (2018). Premature birth, low birth weight, and positive screening for autism spectrum disorder in an early intervention sample. J Dev Phys Disabil.

[56] Mir IN, White SP, Steven Brown L, Heyne R, Rosenfeld CR, Chalak LF. Autism spectrum disorders in extremely preterm infants and placental pathology findings: a matched case–control study. Pediatr Res. 2020. 10.1038/s41390-020-01160-4 32950030

[57] Mohammed HS, Wahass SH, Mahmoud AA (2016). Incidence of autism in high risk neonatal follow up. Neurosciences..

[58] Nagai Y, Nomura K, Uemura O. Primitive reflexes in very low birth weight infants later diagnosed with autism spectrum disorder. Minerva Pediatr. 2020. 10.23736/S2724-5276.20.05784-9 32549029

[59] Persson M, Opdahl S, Risnes K, Gross R, Kajantie E, Reichenberg A, et al. Gestational age and the risk of autism spectrum disorder in Sweden, Finland, and Norway: a cohort study. PLoS Med. 2020;17. 10.1371/journal.pmed.1003207 PMC7508401 32960896

[60] Pineda RG, Neil J, Dierker D, Smyser CD, Wallendorf M, Kidokoro H, et al. Alterations in brain structure and neurodevelopmental outcome in preterm infants hospitalized in different neonatal intensive care unit environments. J Pediatr. 2014;164. 10.1016/j.jpeds.2013.08.047 PMC3872171 24139564

[61] Pritchard MA, De Dassel T, Beller E, Bogossian F, Johnston L, Paynter J, et al. Autism in toddlers born very preterm. Pediatrics. 2016;137. 10.1542/peds.2015-1949 26798043

[62] Rand KM, Austin NC, Inder TE, Bora S, Woodward LJ (2016). Neonatal infection and later neurodevelopmental risk in the very preterm infant. J Pediatr.

[63] Rutkowska M, Bekiesińska-Figatowska M, Kmita G, Terczyńska I, Polak K, Kalisiak M (2018). Neuroimaging results, short-term assessment of psychomotor development and the risk of autism spectrum disorder in extremely premature infants (≤28 GA) - a prospective cohort study (preliminary report). Dev Period Med.

[64] Sharp M, French N, McMichael J, Campbell C (2018). Survival and neurodevelopmental outcomes in extremely preterm infants 22–24 weeks of gestation born in Western Australia. J Paediatr Child Health.

[65] Stephens BE, Bann CM, Watson VE, Sheinkopf SJ, Peralta-Carcelen M, Bodnar A (2012). Screening for autism spectrum disorders in extremely preterm infants. J Dev Behav Pediatr.

[66] Sumanasena SP, Vipulaguna DV, Mendis MM, Gunawardena NS (2018). Beyond survival: 5-year neurodevelopmental follow-up of a cohort of preterm infants in Colombo, Sri Lanka. Paediatr Int Child Health.

[67] Twilhaar ES, de Kieviet JF, Bergwerff CE, Finken MJJ, van Elburg RM, Oosterlaan J (2019). Social adjustment in adolescents born very preterm: evidence for a cognitive basis of social problems. J Pediatr.

[68] Verhaeghe L, Dereu M, Warreyn P, De Groote I, Vanhaesebrouck P, Roeyers H (2016). Extremely preterm born children at very high risk for developing autism spectrum disorder. Child Psychiatry Hum Dev.

[69] Vermeirsch J, Verhaeghe L, Casaer A, Faes F, Oostra A, Roeyers H (2021). Diagnosing autism spectrum disorder in toddlers born very preterm: estimated prevalence and usefulness of screeners and the autism diagnostic observation schedule (ADOS). J Autism Dev Disord.

[70] Yaari M, Yitzhak N, Harel A, Friedlander E, Bar-Oz B, Eventov-Friedman S (2016). Stability of early risk assessment for autism spectrum disorder in preterm infants. Autism..

[71] Yang P, Chen YH, Yen CF, Chen HL (2014). Psychiatric diagnoses, emotional–behavioral symptoms and functional outcomes in adolescents born preterm with very low birth weights. Child Psychiatry Hum Dev.

[72] You J, Shamsi BH, Hao MC, Cao CH, Yang WY. A study on the neurodevelopment outcomes of late preterm infants. BMC Neurol. 2019;19. 10.1186/s12883-019-1336-0 PMC6542031 31146703

[73] Borenstein M, Hedges LV, Higgins JPT, Rothstein HR (2010). A basic introduction to fixed-effect and random-effects models for meta-analysis. Res Synth Methods.

[74] Cheung MWL (2013). Implementing restricted maximum likelihood estimation in structural equation models. Struct Equ Model.

[75] Maenner MJ, Shaw KA, Baio J, Washington A, Patrick M, DiRienzo M (2020). Prevalence of autism spectrum disorder among children aged 8 years-autism and developmental disabilities monitoring network, 11 sites, United States, 2016. MMWR Surveill Summ.

[76] Egger M, Smith GD, Schneider M, Minder C. Papers bias in meta-analysis detected by a simple, graphical test. 10.1136/bmj.315.7109.629 PMC2127453 9310563

[77] Charman T, Gotham K (2013). Measurement issues: screening and diagnostic instruments for autism spectrum disorders - lessons from research and practise. Child Adolesc Mental Health.

[78] Kim SH, Joseph RM, Frazier JA, O’Shea TM, Chawarska K, Allred EN (2016). Predictive validity of the modified checklist for autism in toddlers (M-CHAT) born very preterm. J Pediatr.

[79] Landa RJ, Holman KC, Garrett-Mayer E (2007). Social and communication development in toddlers with early and later diagnosis of autism spectrum disorders. Arch Gen Psychiatry.

[80] Fenoglio A, Georgieff MK, Elison JT. Social brain circuitry and social cognition in infants born preterm. J Neurodev Disord. 2017;9. 10.1186/s11689-017-9206-9 PMC5516343 28728548

[81] Johnson CP, Myers SM, Lipkin PH, Cartwright JD, Desch LW, Duby JC (2007). Identification and evaluation of children with autism spectrum disorders. Pediatrics..

[82] Daniels AM, Mandell DS (2014). Explaining differences in age at autism spectrum disorder diagnosis: a critical review. Autism..

[83] WHO. WHO recommendations on interventions to improve preterm birth outcomes. Geneva; 2015. Available from: www.who.int/reproductivehealth 26447264

[84] Robertson SM. Neurodiversity, quality of life, and autistic adults: shifting research and professional focuses onto real-life challenges. Disabil Stud Q. 2009;30.

[85] Wallace KS, Rogers SJ (2011). Erratum: intervening in infancy: implications for autism spectrum disorders (journal of child psychology and psychiatry (2010) 51 (1300-1320)). J Child Psychol Psychiatry Allied Discip.

[86] Whalen C, Schreibman L, Ingersoll B (2006). The collateral effects of joint attention training on social initiations, positive affect, imitation, and spontaneous speech for young children with autism. J Autism Dev Disord.

[87] Rogers SJ, Williams JHG. Imitation in autism findings and controversies. Imitation Soc Mind Autism Typ Dev. 2006:277–309.

[88] Young RL, Brewer N, Pattison C (2003). Parental identification of early behavioural abnormalities in children with autistic disorder. Autism..

