# Correction to: The prevalence and profile of autism in individuals born preterm: a systematic review and meta-analysis

**DOI:** 10.1186/s11689-021-09402-0

**Published:** 2021-12-24

**Authors:** Catherine Laverty, Andrew Surtees, Rory O’Sullivan, Daniel Sutherland, Christopher Jones, Caroline Richards

**Affiliations:** 1 School of Psychology, University of Birmingham, Birmingham, B15 2TT, UK; 2 Forward Thinking Birmingham, Birmingham Women’s and Children’s NHS Foundation Trust, Birmingham, UK; 3 School of Psychology, Loughborough University, Loughborough, LE11 3TU UK

**Correction to: J Neurodev Disord 13, 41 (2021)** 10.1186/s11689-021-09382-1

In the original publication of this article [1] the following data points were missing from Table 1 (pages 6 & 7) due to a word processing error.

- Mir et al., 2020 – Quality weightings of ‘Autism - 2’ ‘Sample Identification - 1’ ‘Design - 2’.
- Vermeirsch et al., 2020 - Quality weightings of ‘Autism - 2’ ‘Sample Identification - 1’ ‘Design.

The correct Table 1 is shown in the next pages. Two author’s affiliations have been amended to be ‘School of Psychology, University of Birmingham, Birmingham, B15 2TT, UK’. The original article has been corrected.

**Table 3:** Sample characteristics and quality criteria for included papers

Author	Location	Gestational age of sample	Diagnostic tools used	Sample Size	No. preterm individuals scored over the autism threshold	Quality Criteria		
						Autism	Sample Identification	Design
Abolfotouh et al, 2018 [26]	Saudi Arabia	22-23wks (10)	Denver developmental screening test	63	4 (Hyperactive autistic)	1	1	3
		24-25wks (43)						
		26-28wks (52)						
		29-30wks (12)						
Al-Hathlol et al.*, 2020* [27]	Saudi Arabia	< 32 weeks or < 1500 g	Unspecified questionnaire	158	3	1	1	2
Atladottir et al, 2016 [28]	Denmark	24–43 weeks	Diagnosis was retrieved from the Danish Psychiatric Central Register	82,911	1203	1	3	2
Bakian et al, 2018 [29]	Utah	< 37 weeks	Registry of Autism and Developmental Disabilities	4855	112 (ASD)	1	2	2
Boone et al, 2018 [30]	Columbus	< 30 weeks	PDDST-II-DCS	528	190	3	2	3
			ADOS-2	555	24 (ASD)			
Bröring et al, 2018 [31]	Amsterdam	Very preterm (30.2 mean)	SRS, Children’s Communication Checklist, SCQ	57	0	1	1	3
Brumbaugh et al.*, 2020* [32]	Minnesota	< 37 weeks	Medical and educational records	7876	266	1	3	3
Chen et al.*, 2020* [33]	Taiwan	< 32 weeks or < 1500 g	ADOS and ADI-R	324	30	3	2	3
De Groote et al, 2006 [34]	Belgium	< 37 weeks	Autism Diagnostic Observation Schedule-Generic	25	2 (Autism)	2	1	3
De Oliveira Holanda et al.*, 2020* [35]	Brazil	< 37 weeks	MCHAT	40	20	1	1	3
Dudova et al*,* 2014 [36]	Prague	NR	M-CHAT	157	28	3	2	3
			ADOS	33	15			
Gray et al*,* 2015 [37]	Brisbane	very preterm	MCHAT, The CBCL & DASS	97	13 (Autism)	1	1	3
Guy et al, 2015 [23]	East Midlands	32–36 weeks	MCHAT & follow up phone interview	634	92	1	2	3
Hack et al*,* 2009 [38]	America	26 weeks	Parent Child Symptom Inventory	219	8	1	1	2
Harel-Gadassi et al., 2018 [39]	Jerusalem, Israel	31.16 (mean)	M-CHAT	93	25	3	1	3
			ADOS-T	101	8			
Hubert et al.*, 2020* [40]	Poland	GA Mean 27.8	Childhood autism spectrum test	89	5	1	1	3
Hvidtjorn et al.*, 2011* [41]	Denmark	< 37	Public child mental health service	37,283	277	1	2	3
Hwang et al, 2013 [42]	Taiwan	Late preterm = 1078, Later preterm = 28,947, Full-term = 1,104,071	Coded by doctors based on ICD-9-CM	Early preterm = 1078, Later preterm = 28,947	Early preterm = 24, Later preterm = 387	1	2	3
Ikejiri et al*,* 2016 [43]	Juntendo	> 33 Weeks	DSM-4-TR	59	9 (ASD)	1	1	3
Indredavik et al.*, 2004* [44]	Norway	GA mean: 28.8	Interviewed and conclusions drawn according to DSM	56	1	1	2	3
Johnson et al*,* 2010 [45]	UK & Ireland	< 26	SCQ	189	29 (ASD)	1	3	3
Johnson et al*,* 2018 [24]	England	32 wks = 38	MCHAT	638	92	1	2	3
Joo et al.*, 2015* [46]	Korea	24–36	CARS	58	1	2	2	3
Kihara et al.*, 2015* [47]	Japan	GA mean: 27.4	Clinical assessments & DSM criteria	321	35	1	2	3
Klimek et al., 2018 [48]	Poland	28 weeks (Mean)	The Childhood Autism Spectrum Test	86	5	1	1	3
Kuban et al 2009 [49]	US	before 28 weeks gestation	MCHAT	988	212	1	2	3
Kuzniewicz et al*,* 2014 [50]	California	< 24 weeks	ASD evaluation centre	15,696	280	3	2	3
Laerum et al., 2019 [51]	Norway	28.9 weeks (mean)	Autism Spectrum Quotient	59	21	1	1	3
Lean et al.*, 2020* [52]	USA	< 30 weeks	ADOS & Parent report	85	11	3	1	3
Leavey et al, 2013 [53]	California		Diagnostic codes	33,121	213	1	2	2
Lederman et al, 2018 [54]	São Paulo, Brazil	29.5 (mean)	M-CHAT & Autism Behaviour Checklist	60	4	3	1	3
Limperopoulos, et al, 2008 [15]	Boston	NR	MCHAT	91	23	1	1	3
Matheis et al, 2018 [55]	Louisiana	< 37 Weeks	BISCUIT Part 1	687	213	1	2	2
Mir et al.*, 2020* [56]	Texas	< 28 weeks	MCHAT	218	31			
			ADOS & CARS	218	16			
Mohammed et al.*, 2016* [57]	Saudi Arabia	GA 27–33	Clinical assessments & DSM	107	5	1	1	3
Moore et al, 2012 [25]	England	NR	MCHAT	523	216 (Positive result on the MCHAT Autism)	1	3	3
Nagai et al.*, 2020* [58]	Japan	VLBW < 1500	DSM-5 and ADOS	38	10	3	1	3
Persson et al.*, 2020* [59]	Norway	< 37 weeks	Medical records	165,845	3544	1	3	2
Pineda et al.*, 2014* [60]	USA	< 30 weeks	MCHAT	77	19	1	2	2
Pinto-Martin et al.*, 2011* [21]	New Jersey	GA mean:31.2	ADI-R/ADOS	623	14	2	2	3
			SCQ	623	117			
Pritchard et al*,* 2016 [61]	Australia	< 29 Weeks	ADOS-G	15	3	2	1	3
			M-CHAT	169	22			
Rand., et al. 2016 [62]	New Zealand	< 32	DAWBA	102	3	1	2	3
Rutkowska*,* et al, 2018 [63]	Poland	< 28 Weeks	Screening Tool for Autism in Toddlers & Young Children	10	4	1	1	1
Sharp et al.*, 2018* [64]	Australia	22–24 wks	Multidisciplinary team assessment	159	9	1	1	2
Stephens et al, 2012 [65]	NICHD Neonatal Research Network	< 27 weeks	PDDST-II & adapted items from the ADOS	554	Positive screen - 113	2	2	3
Sumanasena et al.*, 2018* [66]	Sri Lanka	< 34	DSM Criteria	39	3	1	2	3
Treyvaud et al, 2013 [14]	Melbourne Australia,	< 30 weeks	DAWBA	177`	8 (ASD)	1	1	3
Twilhaar et al., 2019 [67]	Amsterdam, Netherlands	29.2 weeks (mean)	Social Responsiveness Scale	60	18	1	1	3
Verhaeghe et al*,* 2016 [68]	Belgium	Before 27 weeks	SRS	47	21	3	2	3
			ADOS, and The ADI-R	43	14			
Vermeirsch et al.*, 2020* [69]	Belgium	< 30 weeks	SRS	55	22	2	1	3
			ADOS, ADI-R & clinical information	55	7			
Yaari, et al, 2016 [70]	Israel	24–34 weeks	The AOSI and ADOS-T	99	High ASD risk – 8, Low ASD risk - 91	–	–	–
Yang et al.*, 2015* [71]	Taiwan	Mean BW 1200 g	Diagnostic tools, observations and parental reports	61	2	1	2	3
You et al., 2019 [72]	China	35.5 weeks (mean)	M-CHAT	102	9	1	2	3
