# Supplementary material for: The prevalence and profile of autism in individuals born preterm: a systematic review and meta-analysis
Source: J Neurodev Disord. 2021 Sep 21;13:41. doi: 10.1186/s11689-021-09382-1 (PMC8454175; doi:10.1186/s11689-021-09382-1)
Supplement: Supplementary file 1 — Additional file 1. Supplementary analysis and figures provided for additional clarity. [file 11689_2021_9382_MOESM1_ESM.docx]

| **Supplementary Table 1:** Inclusion and exclusion criteria for title and abstract screening | |
| --- | --- |
| **Inclusion Criteria** | **Exclusion Criteria** |
| Empirical studies | Conference abstracts, conference papers, literature reviews or book chapters |
| Published or available in English | Published or only available in a language not English |
| Peer reviewed | Not peer reviewed |
| Human participants | Studies use animals |
| Title or abstract has ASD reported / referenced | Title / abstract does not report ASD |
| Title or abstract has prematurity reported / referenced | Title / abstract does not report prematurity |
| Sample >5 | Sample <5 |
| Title or abstract refers to categories associated with ASD; Neuropsychiatric disorders, Psychiatric outcomes, Developmental disorders | Title or abstract does not refer to categories associated with ASD; Neuropsychiatric disorders, Psychiatric outcomes, Developmental disorders |
| Title or abstract refers to sample as low birth weight | Title or abstract does not refer to sample as low birth weight |

| **Supplementary Table 2:** Inclusion and exclusion criteria for full text screening | |
| --- | --- |
| **Inclusion Criteria** | **Exclusion Criteria** |
| Reports the number of participants that meet clinical cut off for ASD | Does not report the number of participants that meet clinical cut off for ASD |
| Reports gestational week | Does not report gestational week |
| Data in study were extractable | Not possible to extract data (means) |
| Tools used to assess ASD are reported | Tools used to assess ASD were not reported |
| Sample may have been reported on before, however paper evidences new information on the cohort | Multiple publications from the same sample that do not include new information |


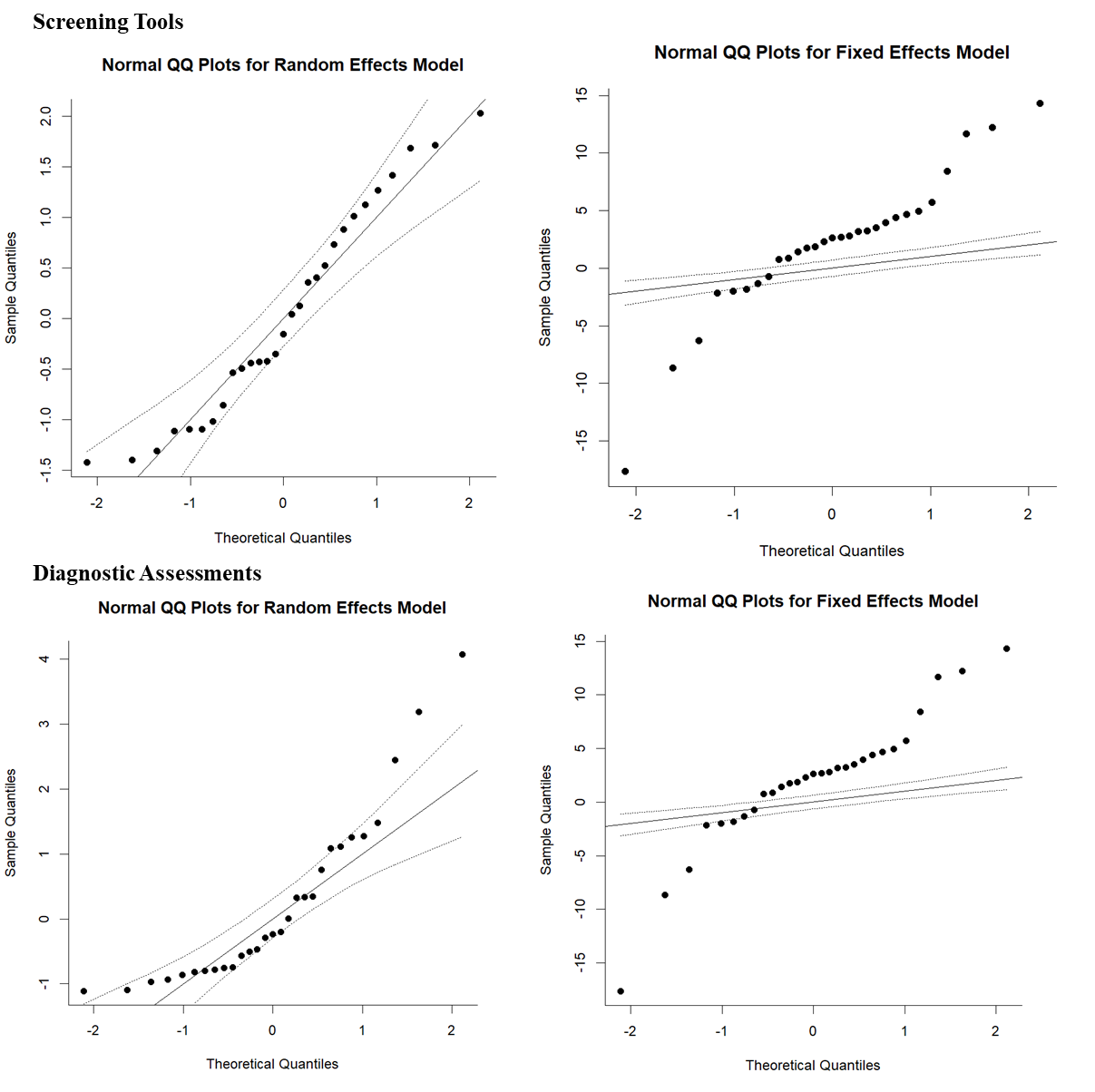


**Supplementary Figure 1:** Quantile-Quantile Plots

The Quantile-Quantile plots show that the observed distribution for the fixed effects model does not conform to the expected distribution prompting the decision to adopt the use of the restricted maximum liklehood estimator.

**Supplementary Figure 2:** The random effects model weighted by the quality effects matrix for pooled prevalence estimates for autism characteristics in individuals born preterm using screening (a) and diagnostic (b) tools.


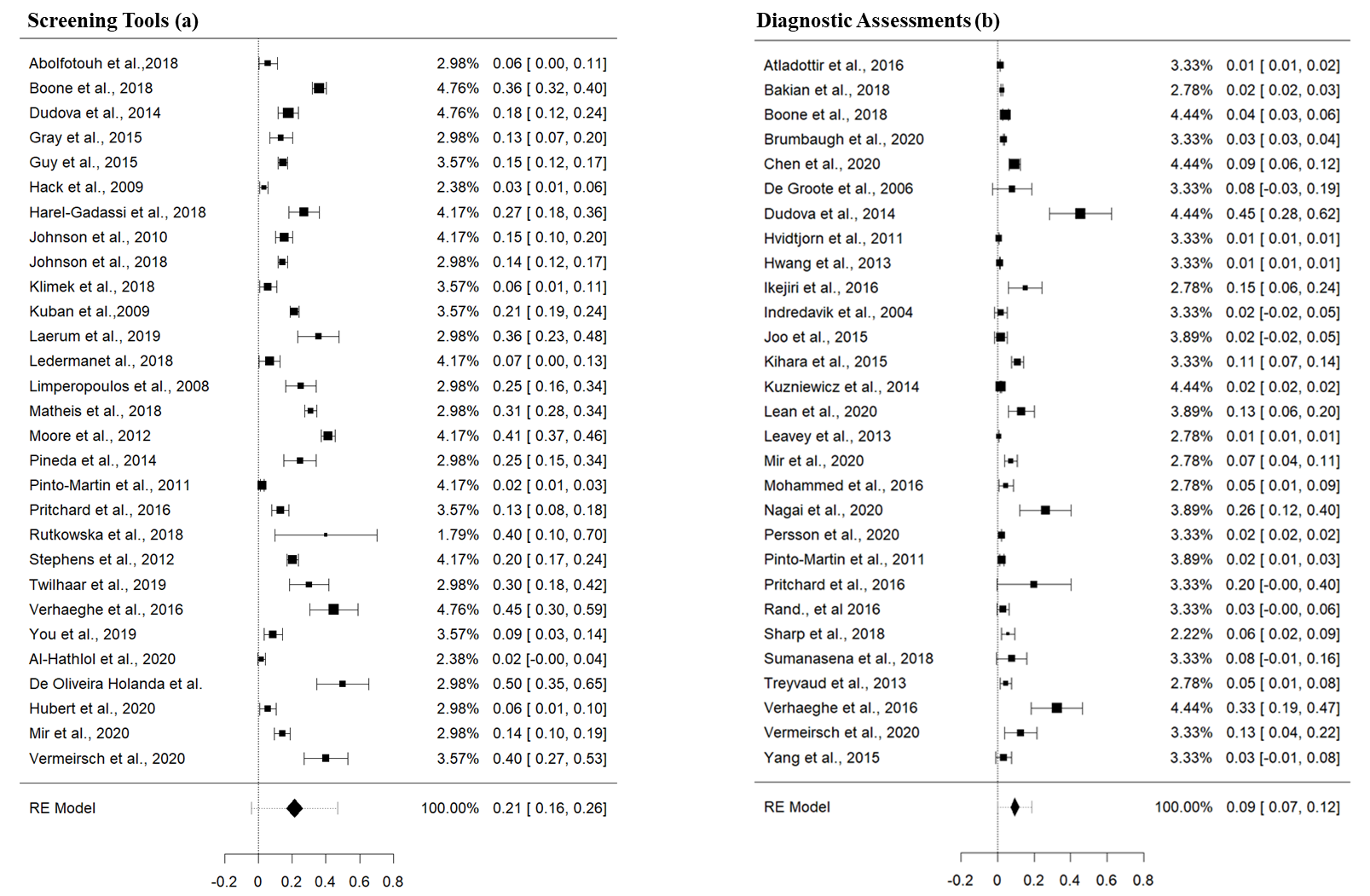


**Supplementary Figure 3:** Pooled prevalence estimate for autism characteristics in individuals born preterm categorised by assessment type using a random-effects model with each study omitted


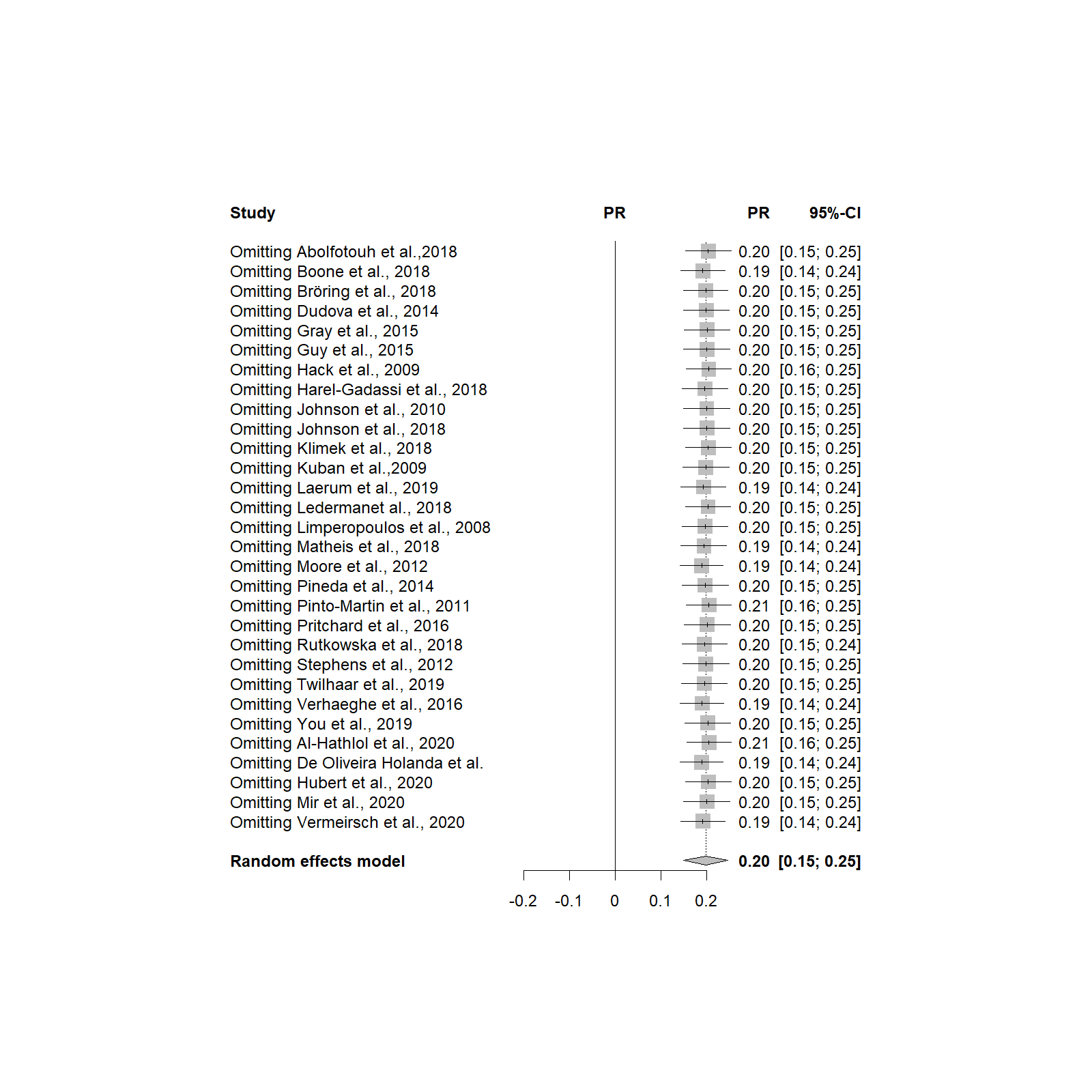


**Screening Tools**


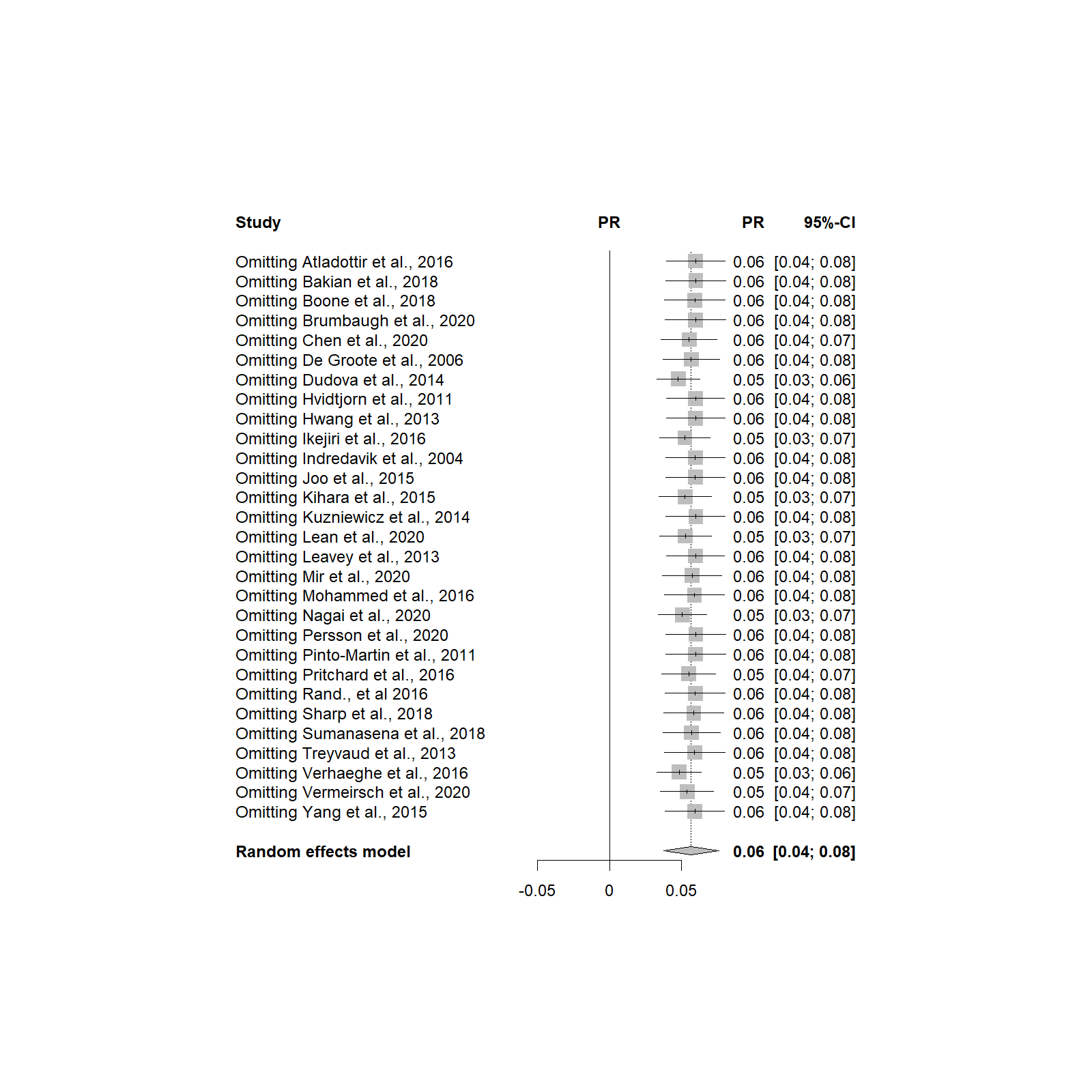

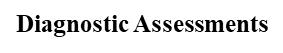


**Impact of influential studies**

To identify any studies that had significantly high impact on the overall effect, a ‘leave one out’ analysis was conducted. Where a study estimate fell outside of the 95% CI for the pooled prevalence estimate, it was considered to have disproportionate effect. Inclusion of such studies did not alter overall prevalence estimates and therefore no studies were removed. To assess studies with large contributions to the overall heterogeneity of prevalence analysis, a Baujat Plot was created ^43^. No studies fell into the quartile of high heterogeneity and impact on effect and therefore no studies were removed. See supplementary materials for results of these analyses.
